# Fibronectin glomerulopathy with monoclonal gammopathy responding to bortezomib plus dexamethasone: a case report

**DOI:** 10.1186/s12882-022-03005-0

**Published:** 2022-11-30

**Authors:** Xiaoli Li, Xueting Qi, Zhigang Ma, Wenhui Huang

**Affiliations:** Department of Nephrology, Gansu Provincial Hospital, Lanzhou, 730000 China

**Keywords:** Fibronectin glomerulopathy, Monoclonal gammopathy, Bortezomib

## Abstract

**Background:**

Fibronectin glomerulopathy is a rare, familial glomerular disease characterized by mesangial fibronectin deposition in the glomeruli. It is caused by the genetic defect in fibronectin and does not involve the activation of the immune system. Therefore, glomerular immunoglobulin and complement staining is generally absent or weak. Monoclonal gammopathy (MG) is an increasing cause of renal lesion, featured by light chain (κ or λ) and/or heavy chain restriction in glomeruli. Herein, we report a case of fibronectin glomerulopathy presenting as strong IgA and C3 immunostaining in renal biopsy, concomitant with monoclonal gammopathy (monoclonal IgA κ).

**Case presentation:**

A 44-year-old female was admitted to our hospital for one-month pedal edema. The serum albumin of 19.6 g/l, and the 24-h urine protein was 15.092 g. Immunofixation electrophoresis displayed monoclonal IgA. The renal biopsy showed the mesangial deposits positive for IgA (3+) and C3 (3+) and also for IgG (2+), IgM (2+), and C1q (2+) IF microscopy. In addition, the staining intensity of light chain κ was slight greater than that of light chain λ. The glomerular deposits were strongly positive by FN by immuohistochemistry. The patient was treated with bortezomib, dexamethasone in combination with cyclophosphamide and gained partial remission.

**Conclusion:**

We present the first FNG patient with strong IgA and C3 immunostaining in the context of monoclonal IgA κ in the circulation. Perhaps FNG, monoclonal IgA κ and immune activation are potentially interplayed and eventually induce renal injuries.

## Background

Fibronectin glomerulopathy (FNG) is an autosomal dominant disorder characterized by glomerular mesangial and subendothelial fibronectin (FN) deposition, with no glomerular immunoglobulin (Ig) or complement (C). The clinical features of FNG include proteinuria, microscopic hematuria and hypertension leading to end-stage renal failure in the second to sixth decades of life, and it was first described in 1987 [1]. In 1992, Mazzucco G et al. identified FN [2] and, then, FNG was recognized as a distinct entity in 1995 [3, 4]. Approximately of 40% of cases are caused by pathogenic variants in the FN1 gene locus at 2q32 [5]. In the other 60% of patients, the causes remained unclear. Herein, we report the first FNG case with strong IgA and C3 immunostaining in renal biopsy concurrent with monoclonal gammopathy (MG).

## Case presentation

A 44-year-old female was admitted to our hospital for one-month pedal edema. She had no remarkable medical or family history.

On admission, her blood pressure was 134/84 mmHg. A physical examination revealed mild edema of the lower extremities. There were no specific findings suggestive of connective tissue diseases.

Initial laboratory data showed decreased hemoglobin of 97 g/L and serum albumin of 19.6 g/l. The renal and hepatic functions were normal. Urinalysis showed proteinuria 3+ and no microscopic hematuria or casts. The 24-h urine protein was 15.092 g, with middle-molecular and large-molecular protein accounting for 62.8 and 37.2%, respectively. Plasma C3 was slightly decreased at 0.74 g/l (normal range 0.79–1.52 g/l), whereas C4 was normal at 0.23 g/l (0.16–0.35 g/l). IgG was 3.41 g/l (7.51–15.6 g/l), IgA was 8.66 g/l (0.82–4.53 g/l), and IgM was 0.66 g/l (0.46–3.04 g/l). C-reactive protein was slightly elevated at 7.5 mg/l (0–5 mg/l). Immunofixation electrophoresis displayed monoclonal IgA κ. A free light chain examination displayed a κ concentration of 595.00 mg/l (0.39–15.10 mg/l) and a λ concentration of 147 mg/l (0.81–10.10 mg/l), resulting in an increased κ to λ ratio of 4.0476 (0.461–4.000). The tests for ANA, anti-dsDNA antibodies, ANCA, anti-ENA antibodies, hepatitis B virus antigen and hepatitis C virus antibodies were all negative. Renal ultrasound revealed normal size and mild cortical hyperechogenicity.

Bone marrow aspiration indicated hyperplastic anemia and 3% plasma cells. The flow cytometry of bone marrow detected 1.22% monoclonal plasma cells which were positive for CD38, CD138, CD117, CD27, CD200 and clonal κ but negative for λ.

**Fig. 1:**
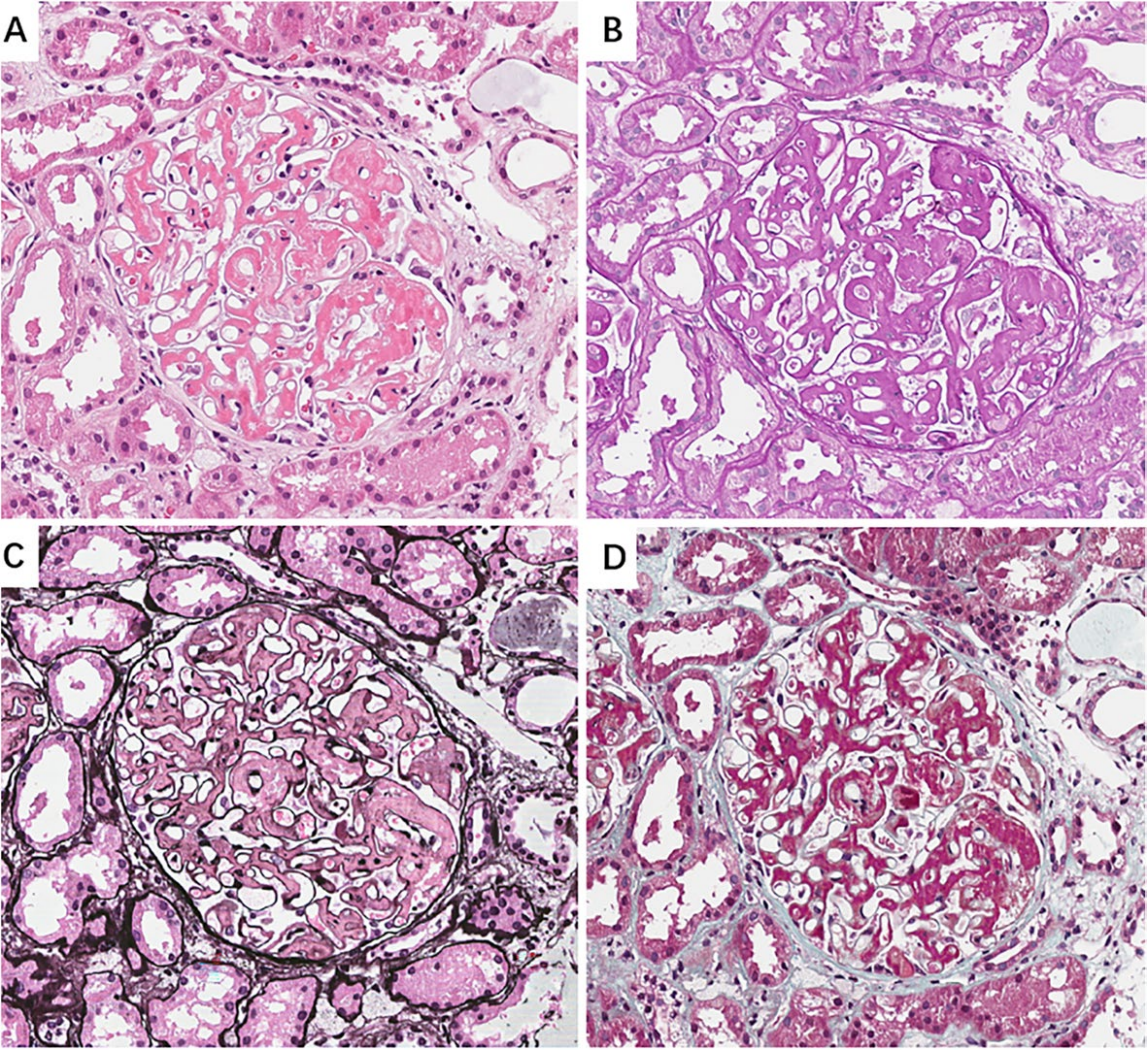
Light microscopic finding (original magnification, × 400). The mesangium was greatly expanded, with minimal hypercellularity, by massive eosinophilic, strongly PAS-positive and silver-negative materials, leading to narrowed or occlusive capillary lumina. **A** Hematoxylin and eosin staining; **B** Periodic acid-methenamine staining; **C** Periodic acid-methenamine silver staining; **D** Masson’s trichrome staining

**Fig. 2:**
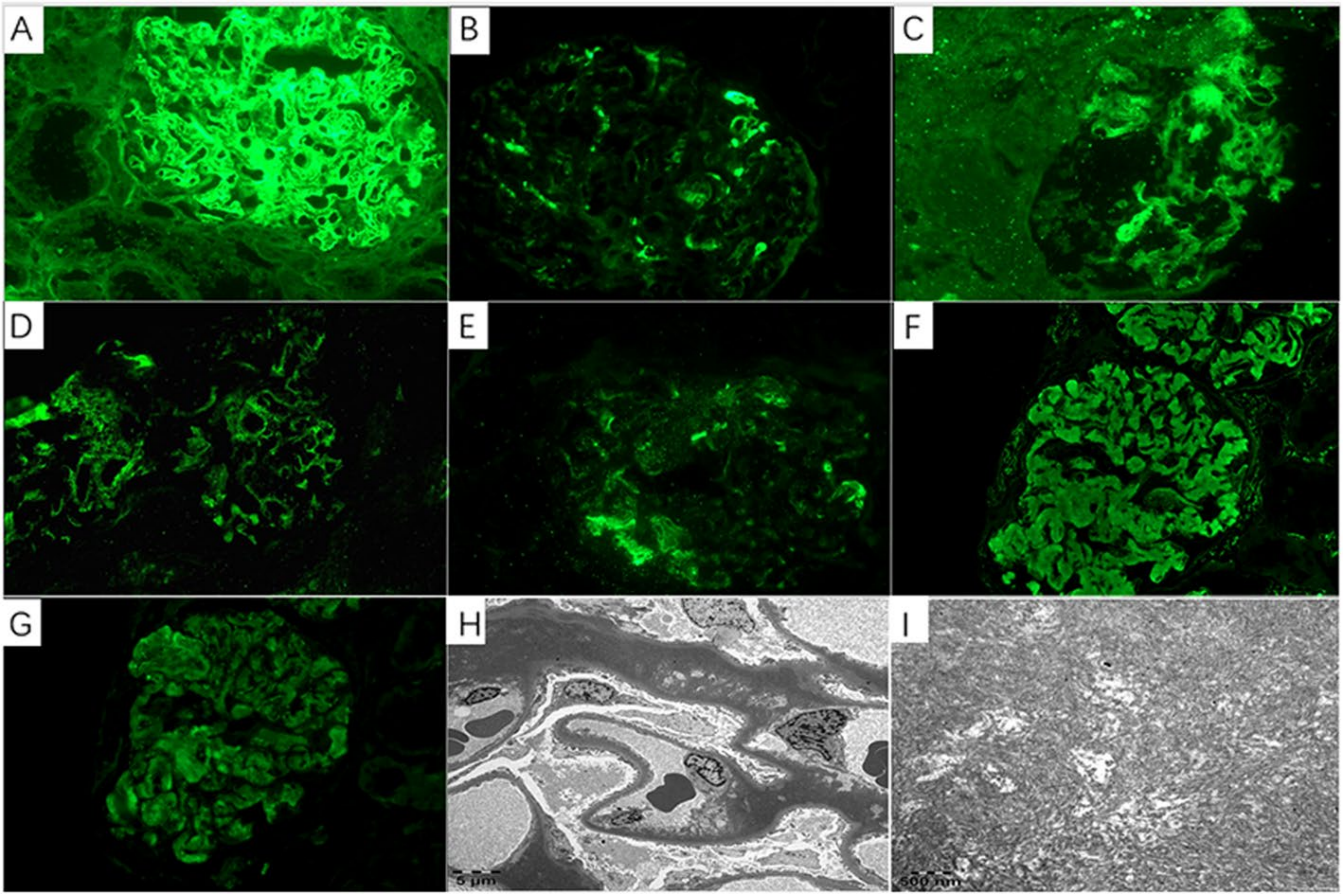
**A**-**G** Under routine IF microscopy, the deposits were stained positively for IgA (3+), IgG (2+), IgM (2+), C3 (3+), C1q (2+), κ (+) and λ (+). **H** Electron microscopy (original magnification, × 3000) displayed massive subendothelial and mesangial electron-dense deposits and minor subepithelial electron-dense deposits, appearing in obscure fibrillary structures. **I** Under electron micrograph at high magnification (× 25,000), these fibrils were generally short, randomly arranged, and measured between 12 and 14 nm in diameter

**Fig. 3:**
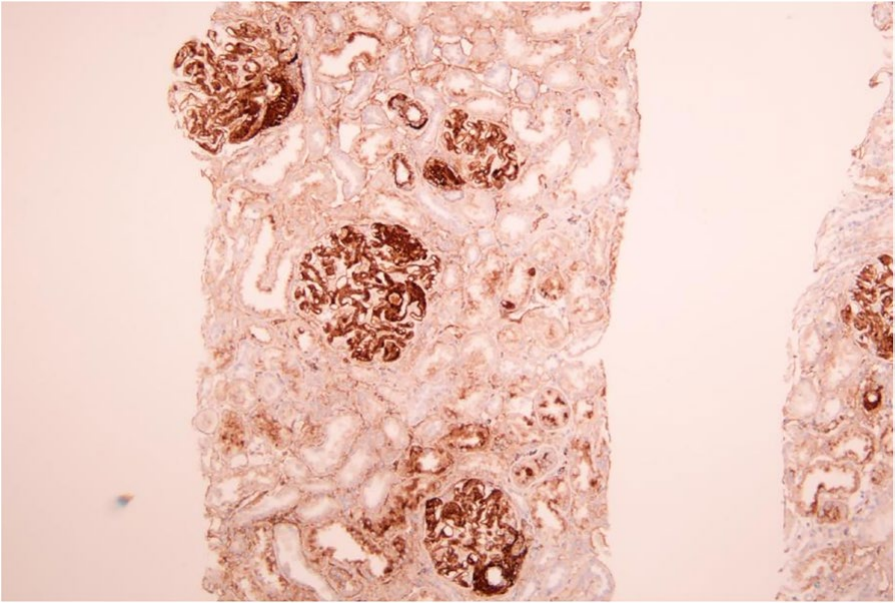
FN immunohistochemistry (original magnification, × 200) showed strongly positive for subendothelial and mesangial deposits

The renal biopsy obtained thirty-eight glomeruli, with two displaying global sclerosis. In other glomeruli, the mesangium was greatly expanded, with minimal hypercellularity, by massive eosinophilic, strongly PAS-positive and silver-negative materials, leading to narrowed or occlusive capillary lumina (Fig. 1). No double contour or crescents were found. There was tubular atrophy in 10% of the cortex and mild interstitial fibrosis. Electron microscopy displayed massive subendothelial and mesangial electron-dense deposits and minor subepithelial electron-dense deposits, appearing in obscure fibrillary structures with diameters of 10–20 nm. The glomerular basement membrane was not thickened. Under routine IF microscopy, the deposits stained dominantly for IgA (3+) and C3 (3+) and also for IgG (2+), IgM (2+) and C1q (2+). Light chains κ was slightly greater than λ (Fig. 2). The Congo staining was negative. Based on the light microscopy and ultrastructural appearance, FN immunohistochemistry was performed using paraffin sections and subendothelial and mesangial deposits were strongly positive (Fig. 3). Therefore, FNG was considered. However, genetic sequencing detected no mutation in FN1.

The patient was given valsartan (80 mg qd) in combination with the BCD regimen (bortezomib 2 mg, dexamethasone 20 mg, plus cyclophosphamide 0.45 mg on Days 1, 4, 8 and 11 every 20 days for 7 cycles and bortezomib 2 mg every month for 6 cycles thereafter).

She was followed up for approximately 3 years and the pedal edema improved gradually. The patient reported no treatment-related adverse effects. The proportion of plasma cells decreased to 0.5%, and the serum IgA level decreased to 0.67 g/L. Monoclonal IgA κ became negative at 7 months post treatment. Two months later, the serum albumin increased from 18 to 25 g/L to 34 g/L, and the 24-h urine protein decreased from 9 to 13 g to 4 g. At her most recent examination, the serum albumin was 40.8 g/l and the 24-h urine protein was 1.54 g. However, her serum creatine increased from 54.6 μmol/l to 102.8 μmol/l (Table 1).

**Table 1:** Details of laboratory tests before, during, and after treatment

	Alb	Scr	Globulin	Hb	IgA	Monoclonal	Gamma	Proteinuria	BM plasma
	(g/l)	(μmol/l)	(g/l)	(g/l)	(g/l)	Ig	globulin (%)	(g/24 h)	cell (%)
Before treatment	19.6	54.6	24.2	86	8.66	IgA κ	6	15	3
1st BCD*4	20	74.1	22.5	118	4.21	IgA κ	11.7	13.3	1.5
2nd BCD*4	21.5	50.1	15.4	95	2.13	IgA κ	–	4.68	–
3rd BCD*4	25.1	62.8	19.8	104	1.92	–	9.9	8.9	0.5
4th BCD*4	23.8	61.2	17.7	83	1.25	IgA κ	9	12	1
5th BCD*4	23	51.8	15.2	94	0.86	IgA κ	7.7	6.95	1
6th BCD*4	27.5	54	17.5	105	0.77	Neg	9.1	9.32	–
7th BCD*4	24.6	39.2	16	99	0.64	Neg	8.6	6.25	–
1st and 2nd BD	34.3	54.7	20.4	113	0.82	Neg	8.9	4.176	–
3rd BD	31	60.8	18.4	96	0.17	Neg	9.7	4.8	–
4th BD	33.5	74.1	18.6	99	–	Neg	10.6	5.238	–
5th BD	36.5	72.2	21	101	–	–	10	–	–
6th BD	40.8	81	18	106	0.72	–	–	1.82	1
3 months after last BD	33.1	68.7	18.9	109	0.8	Neg	8.9	3.21	–
7 months after last BD	41.8	76.4	21.3	128	1.2	Neg	11.6	4.03	–
14 months after last BD	37.96	81.15	17.68	104	1.06	–	10.5	2.49	–
17 months after last BD	40.8	102.8	23.7	99	1.19	Neg	12.3	1.54	–

*Abbreviations*: *Scr* Serum creatinine, *Alb* Albumin, *Hb* Hemoglobin, *BCD* Bortezomib, cyclophosphamide and dexamethasone, *BD* Bortezomib and dexamethasone, *Neg* Negative

## Discussion and conclusion

Herein, we described the first case of FNG with strong IgA and C3 glomerular immunostaining concomitant with MG, which was confirmed by serological monoclonal IgA κ and bone marrow examination.

FNG is caused by the massive mesangial and subendothelial deposits of FN, a high-molecular-weight dimeric glycoprotein of two subunits of 220–250 kDa. FNG has two isoforms: soluble plasma isoform, produced by hepatocytes, and insoluble cellular isoform, locally produced by mesangial cells [6]. In FNG, FN is considered to be plasma-derived because the antibody IST against both forms was strongly stained, but the antibody against the cellular form was weak [4]. The recurrence in renal allografts [7], lack of FN deposits in collapsed or hyperfused glomeruli, [4] and lack of coexpression of other extracellular matrix proteins (tenascin and collagen IV) [4] further supports this opinion. In these patients, the serum level of FN is not elevated, [2] and FN deposits are not detected in other organs (e.g., the lungs, heart, brain, liver, and spleen) [4]. The mechanism of FN accumulation in the glomeruli is unclear. Perhaps there are some abnormalities in FN structure and/or FN-clearing ability of the kidney. A recent study using proteomic analysis found that FN deposits contained fibulin-1 and fibulin-5, which were unique to FNG [8], and concluded that the fibulin-FN complex may play a role in the FNG. In vitro, these FN variants could not bind to heparin on the surface of podocytes and endothelial cells and could not induce endothelial cell spreading and podocyte cytoskeleton reorganization, impairing the kidney. In addition to FNG, FN can also be detected in diabetic nephropathy and lupus nephritis, primarily in the glomerular basement membranes, with relatively similar amounts compared to healthy controls, perhaps due to local secretion by mesangial cells [9, 10].

The diagnosis of FNG is based on histopathologic findings. Light microscopy shows lobular accentuation with mesangial expansion but minimal hypercellularity. The deposits in the mesangium are strongly PAS-positive and silver- and Congo staining-negative, resulting in severely narrowed capillary lumina. Under electron microscopy, these deposits comprise large to massive fibrillary and nonamyloid materials in subendothelial and mesangial spaces with diameters of 12–16 nm. The immunofluorescence staining of Ig or C components is generally negative or nonspecific weakly positive. Immunohistochemistry examination shows strong staining for FN in deposits. In conclusion, the key diagnostic feature of FNG is lobular appearance with massive mesangial FN deposits without Ig or complement deposition. Therefore, the current patient was certainly diagnosed with FNG.

However, this patient also presented with strong glomerular IgA and C3 immunofluorescencent staining, which is not in accordance with FNG. The relationship between FN and Ig is unknown. Some studies have reported the circulatory aggregate of FN with Igs in patients with IgAN and cryoglobulinemia [11, 12], which may deposit in the kidney. Additionally, in some surroundings, FN may be endowed with immunogenicity and activate the immune system in the glomeruli, leading to renal injuries.

The patient also had concomitant MG, which could cause a wide spectrum of kidney disorders with fibrillar deposits featuring light chain (κ or λ) and/or heavy chain restriction in the glomeruli (MG of renal significance). For the patient, the IF intensities of κ was only slightly greater than that of λ, seemingly excluding the possibility that the renal lesion was induced by MG. However, her monoclonal immunoglobulin was IgA κ, while the glomerular fibrillary deposits stained dominantly for IgA and C3, and her renal symptoms were significantly improved by BD regimens, suggesting that the monoclonal IgA κ played some role in the renal injury. The less staining intensity difference of two types of light chains may be caused by deposited IgG and IgM which provide some λ light chain. To identify the relationship between FNG and MG, a PubMed search was conducted using the term “FNG”. Only an 88-year-old patient coexisted with FNG and MG (MIgG κ), with a single C3 deposit (2+) in the glomeruli [13], and the patient died of a heart attack after half a year of hemodialysis and other supportive treatment. No more available information was obtained.

The present patient should be especially differentiated from lupus nephritis, cryoglobulinemia, fibrillary glomerulonephritis, immunotactoid glomerulonephritis, and membranoproliferative glomerulonephritis which can manifest as fibrillar deposits with multiple positive Ig and complement immunostaining [8]. Lupus nephritis is a complication of systemic lupus erythematosus with multiple circulating autoantibodies and multiple organ involvement such as skin, joints, brain, lungs, and blood vessels. Cryoglobulinemia generally affect several organs, especially cutaneous lesion in nearly all patients, and, presents as membranoproliferative glomerulonephritis with subendothelial and/or intraluminal thrombi composed of cryoglobulins, Igs, and/or complement proteins when involving the kidney. Fibrillary glomerulonephritis and immunotactoid glomerulonephritis often have significant hypercellularity and thicker fibrils (10–30 nm diameter and >30-nm diameter, respectively) by electron microscopy [14]. Collagen type III glomerulopathy is characterized by collagen type III deposits with pale PAS staining and whorling and cross-striated fibrils with about 60-nm periodicity [15]. Membranoproliferative glomerulonephritis is a light microscopic pattern of kidney injury, characterized principally by increased glomerular overall cellularity and diffuse thickening of the glomerular capillary walls, forming a double contour to many basement membranes.

There is no standard treatment regimen for FNG. Reportedly, indomethacin [2], mizoribine [16], prednisolone [17] and angiotensin-converting enzyme inhibitors [18] may be somewhat efficacious. Our patient was given ARB in combination with the BCD regimen. The 24-UTP and serum albumin significantly improved 2 months after the monoclonal IgA κ turned negative. It is inferred that MG may play roles in the renal injury, although no pathological evidence of MG-related renal significance was found. The exact mechanism should be explored. The case remains under surveillance, and the association should be further investigated.

In conclusion, we reported the first FNG patient with strong IgA and C3 immunostaining in the context of monoclonal IgA κ in the circulation who partly responded to the BD regimen. Perhaps FNG, MG and immune activation are potentially interplayed and eventually induce renal injuries, but the mechanism needs to be further explored. In addition, FNG should be considered in nonamyloid lobular glomerulopathy, even when immunocomplexes are found in the glomeruli.

## Data Availability

All data related to this case report are within the manuscript.

## Abbreviations

FNG: Fibronectin glomerulopathy
FN: Fibronectin
Ig: Immunoglobulin
C: Complement
MG: Monoclonal gammopathy
